# One for All, All for One: A Mixed Methods Case Study into the Role Organisational and Personal Interests Play on Cooperation in Dutch Integrated Dementia Care Networks

**DOI:** 10.5334/ijic.6424

**Published:** 2022-08-17

**Authors:** Eline Kroeze, Robbert Huijsman

**Affiliations:** 1 Department Health Services and Organisation, Erasmus School of Health Policy & Management, Erasmus University Rotterdam, The Netherlands

**Keywords:** integrated care network, person-centred dementia care, interests, mixed methods, social network analysis

## Abstract

**Introduction:**

Cooperation is key to provide integrated dementia care. However, different kinds of (personal and organisational) interests will affect collaboration in integrated dementia care (IDC) networks. Hence, it is crucial to understand how interests influence relations in IDC-networks in order to shape future policies.

**Methods:**

A sequential mixed methods single case study design was used, in three phases: a Social Network Analysis (SNA) based on data from questionnaires (n = 24–26), explorative interviews with network partners (n = 14) and a focus group with network coordinators (n = 7) to explore the conceptual generalisability of the single case of the IDC-network.

**Results:**

The SNA revealed that highly connected organisations were often established care organisations that deliver case management, while smaller care organisations or welfare organisations tend to be less connected. Care-related, strategic, and financial interests influence participation of organisations in the IDC-network, while personal intrinsic motivations determine a representative’s contribution to the network. Especially conflicting interests strongly influence the network structure.

**Discussion and conclusion:**

We conclude that conflicting interests in IDC-networks stand in the way of reaching the collective goal of an IDC-network, i.e., optimising the wellbeing of people with dementia and their informal caregivers in the region. Thus, IDC-networks should act to manage, resolve and prevent disputes arising from conflicting interests.

## Introduction

Since 2004, the Dutch Ministry of Health, Welfare and Sport actively commits to improving Dutch dementia care. With the introduction of the National Dementia Strategy 2021–2030 this commitment intensifies the coming decade [1]. This intensification is needed as dementia will continue to have a big impact on the Dutch society; forecasts show that the number of people with dementia will increase from now 280.000 to 520.000 in 2040 [2]. The growing needs for dementia care combined with budgetary and labour market pressures make cost containment measures essential; resulting in policy trends which are aimed at reducing and delaying institutionalisation. As dementia care services are not only provided by various professionals working in different organisations, but also financed by different financial systems and policy sectors (see Appendix 1), much fragmentation is present in Dutch dementia care.

Integrated care is considered a key strategy to overcome care fragmentation. Service models for integration have been developed and evaluated in various countries for different subpopulations [3]. Older people with dementia may particularly benefit from integration as their care needs are complex, continuously changing and often required over a longer period of time [4]. The WHO defines integrated care as “the delivery of a continuum of care, designed to meet multidimensional needs of the population and the individual, by a coordinated multidisciplinary team of professionals” [5]. This increased collaboration and coordination between professionals and care organisations across the entire continuum is expected to lead to better patients’ care experiences and a greater health of the population, while also reducing healthcare costs per capita (‘the Triple Aim’) [6,7]. Integration of care can be stimulated by interventions on different levels of organisation, ranging from interventions on the micro-level (e.g. case management), to establishing collaborative inter-organisational relations on the meso-level (e.g. integrated care networks) and system-level interventions on the macro-level (e.g. bundled payments). Valentijn [8] argues that fostering integration across all these levels is key for achieving integrated care.

As interorganisational networks are flexible and ensure pooling of competencies, they are regularly used mechanisms to promote integration on the meso-level of care organisation [9]. Working in interorganisational networks enhances learning, and is likely to result in higher quality services for patients and more efficient use of resources [10]. Gazley & Guo [11] indicate that research has been done on the “why’s and what’s” of collaboration in interorganisational networks, but that research on internal processes of partnership activity is lacking. They argue that more mixed methods studies on collaborative service delivery are needed to get a better understanding of these processes. This research adds to the latter by providing not only an insight in the relationships present in an integrated dementia care (IDC) network, but, more importantly, to understand how personal and organisational interests affect interorganisational cooperation in these networks.

## Theoretical framework

### A model for integrated care

**Figure 1:**
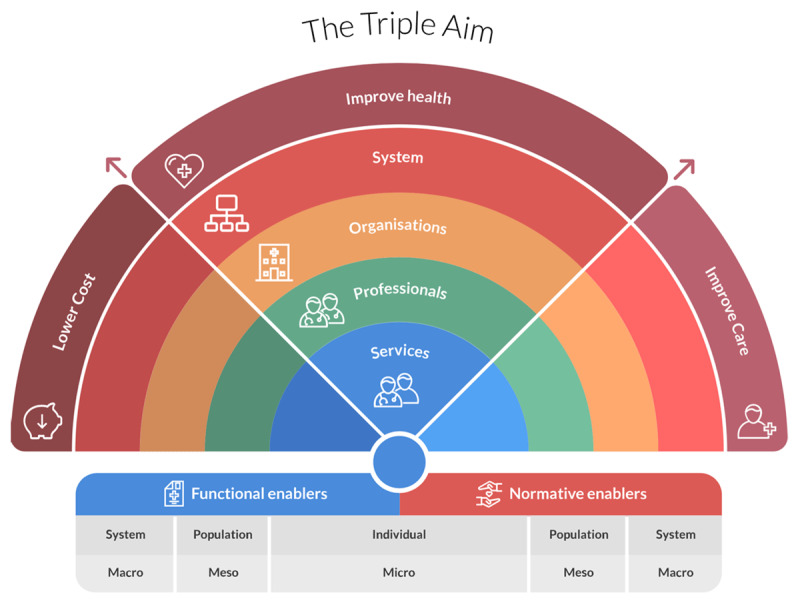
Valentijn’s Rainbow Model of Integrated Care [33].

Valentijn’s Rainbow Model of Integrated Care (RMIC) [12] functioned as the theoretical basis for understanding integrated care and its multi-layered nature. The RMIC (see Figure 1) provides a theoretical framework for describing the different types of integration aimed at the service level (e.g. self-management, case management), professional level (e.g. multidisciplinary care, continuity of care), organisational level (e.g. regional IDC-networks, managed care programs) and system level (e.g. healthcare policies and regulations). Enablers, which are necessary to integrate care at the various levels, can either be functional (e.g. IT, financial incentives) and normative (e.g. cultural values, a shared vision). The RMIC shows that integration is not an isolated incident that takes place at one single level. Different processes of integration at and across levels of integration enhance integration at one single level and vice versa. In this research, the focus is on integration on the organisational (meso-) level in the form of a network structure.

### Network coordinator

**Figure 2:**
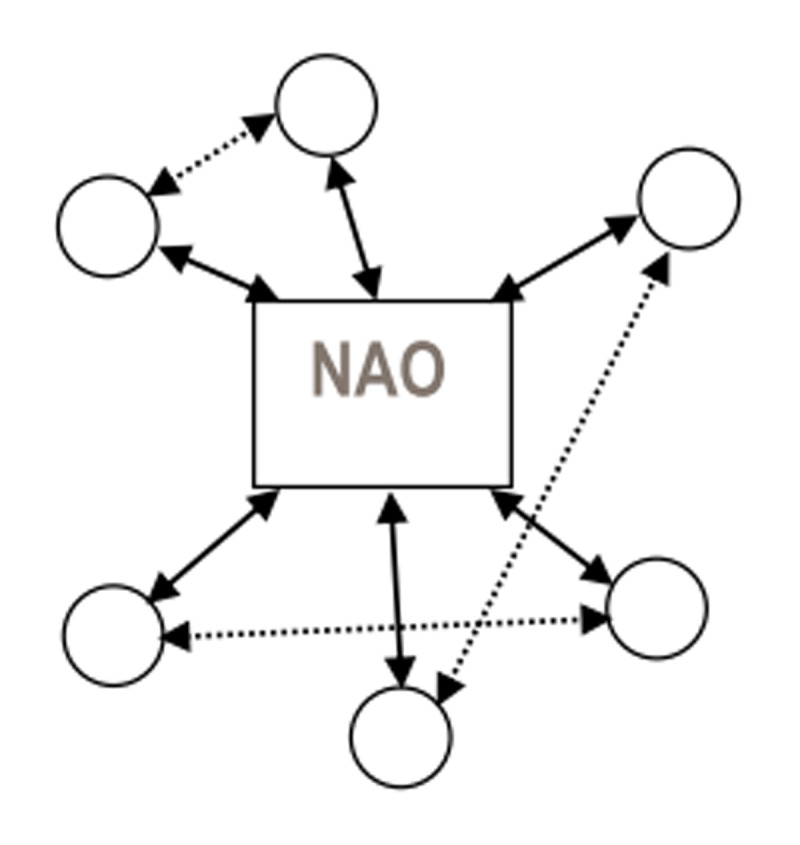
NAO governance structure [13].

The governance structure of the Dutch IDC-networks can be categorised as a Network Administration Organisation (NAO). A designated and separate entity is created to manage and coordinate the activities in the network: the network coordinator [9]. This person is solely focused on network governance and not a member of one of the network partners. Figure 2 graphically depicts the NAO-model of governance. Network partners may interact with each other, but many activities are usually coordinated through and by the network coordinator. Provan and Kenis [13] argue that NAO-models of governance are suitable for networks with moderate to high trust, moderate to many participants, moderately high goal consensus and a high need for network level competencies.

The role of the network coordinator in Dutch IDC-networks is to connect relevant stakeholders for dementia care in the region and to stimulate them to take on their tasks with respect to providing integrated dementia care. The idea is that their activities lead to an increase in the degree of integration in the network; from Leutz’s [14] ‘linkage’ category of integration (which entails that providers work together on an ad hoc basis) to ‘structured coordination’ (where providers share information and collaborate while organisations uphold their own service responsibilities and funding). Furthermore, the network coordinator is not only active on the organisational level, but also on the other levels of the RMIC. He/she moves at and across these levels (e.g. clinical, professional, organisational & system) to connect the right actors at the right time in order to provide integrated dementia care in the region. Also, they function as the link between national and regional policy levels.

### Interests

Integrated care is a collective process and its implementation and execution depends on collaboration between different actors, ranging from professionals, organisations, governments and health insurers [15]. And although these actors collaborate to provide better care, they may have different interests that guide their actions and relationships within the IDC-network [16]. An interest is something ‘of importance for’ a particular person or organisation. When things ‘of importance’ are different and incompatible, conflicting interests arise, which might appear at and across the different levels of integration (i.e. between network partners, the network itself and stakeholders at the system level) [17]. Fabbricotti [18] distinguishes three categories of interests in the health care domain: care-related interests, financial interests and strategic interests around symbolic, reputational or legitimacy matters.

## Methods

### Study Design

The study has an explanatory mixed methods single case study design in three phases: 1) a Social Network Analysis (SNA) based on data from questionnaires (n = 24–26), 2) explorative interviews (n = 14) and 3) a focus group with network coordinators (n = 7). This mixed methods design is suited to acquire a thick understanding of the network structure and the internal processes of collaboration. Wald [19] argues that this type of design is particularly fitting for complex research questions where “*individual meaning, perception, frameworks of relevance and additional context factors play an important role*”, which is definitely the case in this research about (the effects of) interests on interorganisational collaboration.

### Case selection

To generate a detailed understanding of an ICD-network, the study is designed around one single case in order to access and analyse the extensive and detailed data that are needed. As the research is carried out under strict time constraints, a multi-case study was not feasible. However, a focus group (phase 3) has been held to explore the (conceptual) generalisability of the results. In the selection of the IDC-network that would function as the single case for this study, the willingness of the network coordinator and partners to cooperate in research were particularly important. ‘Dementie Twente’ is an active network with relatively active network partners. This reputation, together with the already available contacts in the region Twente, were the deciding factor for choosing network ‘Dementie Twente’ as the case to be studied.

‘Dementie Twente’ is an interorganisational partnership in the East of the Netherlands focused on facilitating cooperation and information-exchange between 42 organisations (see Table 1) of which 27 are active in the care domain, 9 in the social domain, 5 in the medical domain (2 hospitals, 1 mental care organisation and 2 GP care groups) and 1 as patient representative. The network’s mission is to “*increase the quality of life of people with dementia in our region*”. Two independent network coordinators have the explicit task to coordinate the partnership relations within the network and to facilitate in transferring national policy to the regional context. In the region Twente, case management is provided according to the linkage model; multiple case management providers are active, and the case managers act as mediator between the client and involved care, social and medical organisations.

**Table 1:** Network partners and respondents.


NETWORK PARTNERS’ CHARACTERISTICS			NETWORK PARTNERS’ PARTICIPATION IN RESEARCH		
	
CODE	DOMAIN	DELIVERS CASE MANAGEMENT	FILLED-IN SNA- QUESTIONNAIRE	INTERVIEW RESPONDENT (CODE)	POSITION OF RESPONDENT

A	Social				

B	Other		X	X (B)	Chairman of the Board

C	Care		X		Director

D	Care				

E	Care	X	x*	X (E)	District nurse

F	Care	X	X	X (F)	Manager care

G	Care				

H	Care				

I	Care	X	X		Casemanager

J	Care				

K	Care		X	X (K)	Advisor informal care

L	Social		X	X (L)	Director

M	Medical				

N	Social		X		Elderly counselor

O	Care				

P	Care		X	X (P)	Director

Q	Care	X			

R	Social			X (R)	Director

T	Care		X		Location manager

U	Care	X	X		

V	Care	X	X		Senior advisor district nursing

W	Care	X	X		

X	Medical				Director

Y	Medical		X		Internist geriatric medicine

Z	Care	X			

AA	Social				

BB	Social		X	X (BB)	Coordinator

CC	Care				

DD	Social		x*	x (DD)	Course coordinator

EE	Social				

FF	Medical		X	x (FF)	Coordinator care programs

GG	Care		X		Director

HH	Care	X	X	x (HH)	Regional manager

II	Care	X	X		Specialised nurse

JJ	Social		X		Elderly counselor

KK	Care				

LL	Medical		X		Manager

MM	Care	X	X	x (MM)	Director

NN	Care	X	X		Casemanager

OO	Care	X	X		Casemanager

NAO (=S)	–	–	X	2X NAO1, NAO2	Network coordinators


* Only answered the information-exchange questions.

### Data collection and analysis

#### Phase 1: Social Network Analysis

A SNA-analysis was conducted based on the network data provided by 24–26 representatives of network partners (see Table 1) and was carried out in order to analyse the strengths and directions of the network partners’ relationships to other network partners. During the SNA a socio-centred perspective was taken: the overall network structure was assessed, central and isolated actors were indicated, and patterns of ties were analysed and defined as concepts like ‘clusters’ [20]. Based on the theoretical framework and the functions of an IDC-network the SNA was applied to three levels of actor exchanges:

1. Information-exchange at the organisational level (meso): ties that represent information-exchange on the organisational level.
2. Organisational cooperation (meso): collaborative ties between two organisations on organisational level.
3. Cooperation at client level (micro): collaborative ties between two professionals that work together to provide dementia care for a specific person.

SNA-questionnaires were sent to representatives of network partners (n = 42) to be filled out online (using Qualtrics XM software). The higher the response rate, the more complete the visualisation of the network is. Hence, the questionnaire was designed to be concise and not time-consuming for network partners to fill in. The questionnaire included 11 questions; 6 on organisation and representative characteristics; 3 questions in which respondents filled in their frequency level of contact (never, occasionally, regularly, very often) on the three levels of actor exchanges as explained above; a question on whether conflicting interests have an influence on this frequency of contact; and a final question on the perceived role of the network coordinator in facilitating information sharing, cooperation and resolving conflicts surrounding interests.

The SNA was carried out separately for the three levels of actor exchanges. First, the data were extracted from Qualtrics XM to Microsoft Excel. Then, edge lists and node lists were derived from the dataset, which were then imported in R Project. To develop Social Network Graphs and compute network- and node-level metrics, codes were used from the ‘*igraph’, ‘EconGeo’, ‘DT’, “htmlTable’, ‘networkD3’, ‘sna’, ‘ndtv’, ‘visNetwork*’ and ‘*RColorBrewer*’ packages (see Appendix 2 for the full SNA-codes).

Once the SNA was carried out and a visualisation of the network and measures like degree and centrality were available, purposeful sampling of respondents for the semi-structured interviews started.

#### Phase 2: Semi-structured interviews

A representative sample of network partners for the qualitative part of the study was selected, based on the following criteria:

- Respondents from organisations that were highly connected, moderately connected and poorly connected.
- Respondents from care, social welfare, medical and patient organisations.
- Respondents from organisations with a distinctive position in the network.
- Respondents that did not fill-out the questionnaire.
- Respondents from the Network Administration Organisation (NAO), i.e., the network coordinators.

In this way we selected 14 respondents for semi-structured interviews. The topic list was developed cyclically based on the theoretical framework and SNA-results. The interviewees were asked to reflect on their position in the visualised network, in order to ensure triangulation of data. But more importantly – following Hammersley’s [21] remark that “*the feature of language is its capacity to present descriptions, explanations and evaluations*” – this phase of the study was focused on giving an explanation for the structure of the IDC-network as presented by the SNA. The interview topics were aimed at exploring the motivations of organisations and their representatives behind their current position in the networks, with a special focus on the role of interests. Furthermore, interviewees were asked on their views on the role which the network coordinators (should) have in the IDC-network. Saturation was a guiding principle during the collection of the data from interviews; no more interviews were held when the collection of data did not add any values anymore.

The interview data were analysed by means of axial coding, deploying the program Atlas.ti. The coding procedure consisted of three steps. In the first step of open coding, different segments were established by assigning codes and then ordering and grouping them. In the second step, the found codes of categories were related. Thereafter, selective coding was used to identify the most important categories.

#### Phase 3: Focus group

While this mixed methods single case design allowed for a thick understanding of one dementia care network, a focus group with 7 network coordinators (codes: FR1-FR7) was held to explore the (conceptual) generalisability of the results on the findings at the level of the IDC, but particularly on the level (and the role) of the network coordinator. The focus group was held online via MS Teams (due to the COVID-19 pandemic). Participants were selected by the researchers; in this process particular attention was given to diversity in terms of network characteristics and geographical position (see Figure A in Appendix 1). One of the researchers chaired the meeting and presented statements and preliminary results for discussion. An assistant paid attention to nonverbal communication during the focus group and could allow participants to take the floor.

Data were generated by discussion and interaction between group participants; they presented their own views or experiences regarding the statements and SNA-pictures shown, but also listened to other participants’ arguments. This listening, followed by reflections, made them consider their own standpoint further. This particular process is the added value of conducting a group-interview in a focus group; it ensures that individual responses become sharpened and redefined, and move to a deeper and more considerate level [22]. The transcript of the focus group was analysed by means of axial coding in Atlas.ti.

## Results

### SNA-results

**Figure 3:**
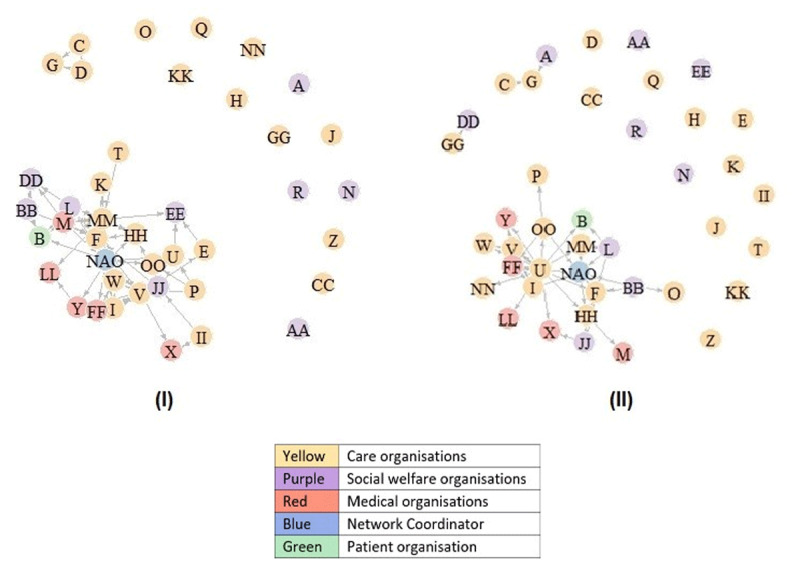
Social Network Graphs: Very often information exchange **(I)** & interorganisational cooperation **(II)**.

The SNA-analysis reveals the individual positions of network partners in the IDC-network and with whom and how often each network partner has exchanges (information exchange, collaboration on organisational level or cooperation at client level). Social Network Graphs were developed for each theme on different frequency levels of cooperation or information exchange. In Figure 3, the Social Network Graphs with links representing ‘very often’ information exchange and organisational cooperation are shown. The Appendices 3–5 present the full SNA and all other Social Network Graphs. A link is present from one actor to another if an actor indicated in the SNA-questionnaire to cooperate (or exchange information in the information-exchange network) – on that frequency level or on a higher frequency level. The distances of the arrows are arbitrary.

Generally, small care organisations and social welfare organisations are poorly connected, while highly connected organisations are established care organisations which deliver case management. A connection between two organisations is more likely when they operate in the same area or field, have the same working culture and if directors or employees already know each other personally. The network on client level is quite different from the network at organisational level. The former is denser, which means that organisations seem to cooperate more often on client level matters. Also, the position of the network coordinators (NAO) is quite different on client level; while they are the most central entity in the informational and organisational networks, they are not acknowledged by other organisations as central in the cooperation at client level network.

### Semi-structured interviews results

Following the interviews with representatives of network partners and coordinators, access to information, knowledge, expertise, support, and regional contacts are the main reason for becoming a network partner. Financial stimuli as creating brand-awareness, increasing the number of referrals to one’s organisation and being stronger together in the bargaining process are other reasons for membership. Strategic stimuli for membership of the IDC-network centre around creating more awareness about dementia in society and (fore)seeing the need of cooperation to deal with future challenges.

As reason for a poorly connected position of an organisation in the network, the tendency to first have things sorted out in one’s own organisation and clients can be pointed out. For organisations that only operate locally, this means that local collaboration could be more essential than regional cooperation initiatives like the IDC-network. Nevertheless, these locally oriented organisations stressed that sharing knowledge and expertise on the regional level is still of considerate importance for them:

“*It is for our social care provision not directly of importance to be active on the regional level. It is, however, important to have local partners and to have sound agreements and successful coordination with them, so that we know how to find each other*.” (L)

Other conflicting interests between the IDC-network itself and the organisations do not emerge from the interviews. The reason seems to be that everyone shares ultimately a common goal, i.e., optimising the wellbeing of people with dementia and their caregivers in the region.

Conflicting interests between organisations were found to have a significant influence on how a partner is positioned in the network. The idea that “*Every client or patient is money*” leads to a system in which care organisations see each other as competitors, which hampers relations at the organisational level. Respondents argued that this competitive environment stems from the Dutch health system:

“*This is not because people don’t want to cooperate, but it is due to the Dutch laws and regulations that these conflicting interests exist*.” (R)

Additionally, conflicting visions on how to deliver good care hinder cooperation at the organisational level; often these are rooted in different visions on the job of ‘case manager’:

“*Case management means different things to different organisations. […] One organisation provides case management as a separate function and for the other organisation it is a part of the tasks of a district nurse*.” (DD)

These clashing visions lead to a weak or no link between two organisations in the IDC-network as a situation of (partial) radio silence between the management of parties arises. Nevertheless, radio silence on the management level does not necessarily translate to poor cooperation on client level (a finding which the SNA-analysis confirms).

The underlying motivations of representatives of network partners for working in dementia care range from personal experiences with someone with dementia in their close circle to frustration with the current state of social awareness or cooperation in dementia care. Following from the interviews, one can conclude that these intrinsic motivations play a role in how active a representative is in the IDC-network. However, the level of activity also depends on the attitude of the manager; if the person easily engages in contact and cooperation and is concerned about his/her reputation in the region. Some directors like to have a say in regional matters or like the feeling of ‘being important’ in the region.

A clash between personal interests of representatives and the interests of their organisation or the IDC-network does not appear to occur. Interviewees explained this phenomenon again by the fact that at all three levels the common goal is to optimise the wellbeing of people with dementia and their informal caregivers. Different interests between executional layers (i.e. representative, organisation and network) may exist, but a clash does not arise since:

“*… it is all about the client and the caregiver […], so that is what you are doing it for. You don’t have a goal for your own organisation; it is all derived from this*.” (HH).

Most interview respondents argue that the network coordinators are vital to keep the network moving. The following responsibilities of the network coordinators with respect to information exchange were deemed most important: providing concise and unambiguous information, translating regional knowledge to the local context and being the bridge between regional policy and knowledge. Moreover, they argue that the network coordinator should be up to date about every network partner and regional project, in order to connect the dots for interorganisational cooperation. Respondents emphasised that taking initiative, being facilitating, and thinking transcendently are important skills of a network coordinator in order to facilitate collaboration at organisational level.

The interview respondents agree that the IDC-network should resolve conflicting interest between network partners, as these could stand in the way of fulfilling the ultimate goal of optimising the wellbeing of the target group. However, there is no clarity or agreement in the IDC-network ‘Dementie Twente’ on who is responsible for settling these disputes. According to the majority of the interview respondents, the network coordinators seem to be the right entity to act as mediator in these situations, but there is one caveat; they should behave impartially. Next to being impartial, the network partners should always shift the focus of the discussion to the client (and his/her caregiver) when managing conflict:

“*I think that the network coordinators have a big role to play as mediator, because they do not only look at the interests of a particular organisation, but especially at what is in the interest of the client. The art is then, for them, to use these organisational interests in order to do what is good for the client*.” (HH)

Finally, they argue that a strategy is needed to prevent (conflicting) interests to even lead to a situation in which they hamper cooperation in an IDC-network. The responsibilities of the network coordinators are then extended with implementing, evaluating, and updating this strategy.

### Results focus group

The network coordinators participating in the focus group see similarities between the structure of ‘Dementie Twente’ and their own IDC-networks. However, they stressed that poorly connected organisations are just as important for the network:

*“These organisations are often involved ‘on a task focused’ form of cooperation. So it seems that they are more peripheral or less active; but that does not need to be the case*.” (FR1)

Also, they remarked that it varies per IDC-network how active the network coordinator is in cooperation on client level. This mostly depends on how case management is organised in an IDC-network, i.e., centrally or not. However, as the focus group concluded, hardly any network coordinator in the Netherlands has direct contacts with clients; they are at most facilitating in assigning case managers to clients or linking case managers with general practitioners.

The networks coordinators agree that interests have a considerable influence on the relationship-structure in their networks; especially conflicting interests and clashing visions between organisations. They also mention that different visions exist between the system-level (national policy) and organisations active in the IDC-networks with respect to the job profile of a case manager:

*“In many organisations there is a shortage of district nurses. And what organisations then often want, is that district nurses follow the training for case management, but that they also keep working as a district nurse. And that is contradictory with what we nationally would like to see; that being a ‘case manager’ is a separate function and that 24 hours per week should be dedicated to case management, as these hours are much needed.”* (FR3)

All participants agree that the network coordinator has an important role as mediator in the case when conflicting interests arise:

*“I think that this is the core of our profession; that is where we are working for during the day. If there would be no conflicting interests, we would become unnecessary.”* (FR1)

However, in order to be able to function as a mediator, impartiality – which is inherent to the function of a network coordinator – is of great importance and value. Hence, the focus group concluded that a network coordinator should always keep working on impartial profiling towards the rest of the IDC-network. Only then it is possible to maintain good relations, to discuss with all parties their problems and interest, and to get everyone on the same page. The focus group argued that this impartiality does not hold for the following exception: a network coordinator should always be at the side of the client, with a strong focus on the IDC-network’s ultimate goal: organising care in such a way that it improves the wellbeing of the person with dementia and his/her caregiver.

## Discussion

This study showed that working together as organisations across the entire continuum of care is challenging, as Kodner and Spreeuwenberg [23] argued. Organisational and personal interests have a considerable influence on the participation of a certain organisation in the IDC-network. Especially conflicting interests (e.g., financial interests and clashing visions) between organisations were found to have a significant influence on how a partner is positioned in the network.

The RMIC framework was helpful in understanding how the perspectives of network representatives, organisations and the network coordinators relate to different dimensions of integration and how various processes of integration at and across levels of integration enhance integration at a single level and vice versa. The degree of cooperation and thus integration in the IDC-network dementia Twente falls between Leutz’s category of ‘linkage’ and ‘structured coordination’ [14]. Highly connected organisations (in general care organisations which deliver case management) work together in a structured way while they uphold their own service responsibilities and funding. Less connected organisations work on an ad hoc basis together with partners in the network. It was found that Fabbricotti’s [18] care-related, financial, and strategic interests were indeed clearly present at the organisational level, but lesser so at the personal level of representatives. However, not merely interests, but especially conflicting interests between organisations appear to have a considerable influence on the structure of an IDC-network. When respondents mentioned tensions between two organisations due to conflicting interests, a collaboration link on organisational level was often not found in the SNA.

Currently, there are no other studies that also used a SNA to analyse the structure of regional IDC-networks. Neither could we use SNA-studies on other types of integrated care networks to compare the structure of an IDC-network, as these focus on professional or social networks, on other foci of integration, or are conducted in different countries [24,25,26,27]. Thus, the findings related to the exact structure of this IDC-network cannot be compared to previous research. However, like Zonneveld et al. [28], this study also pointed out that existing personal relations between managers are the determinant of interorganisational collaboration in Dutch IDC-networks. This corresponds with Gulati’s and Garguilo’s [29] theory of ‘relational embeddedness’; actors who already maintain relations, are more likely to form a network together. Furthermore, our findings show that a link between two actors is more likely when they have matching interests. This resembles with the idea that interests are ‘the engine’ of collaboration processes: interests are the reason why organisations seek each other out, as both parties possess capital that is of value for the other [30,31,32].

The mixed methods design allowed for data triangulation with respect to the network structure of ‘Dementie Twente’ as respondents evaluated the developed SNA during the interviews. They recognised the general structure of the Social Network Graphs, including the interview respondents that did not answer the questionnaire before. Some comments were made about missing links, hence the SNA was updated. However, one has to be conscious of the fact that representatives filled out the survey from their own perspective and cannot precisely estimate with whom and how frequent their employees have contact with employees of other organisations. Especially managers of large organisations indicated that it was difficult to explicate cooperation frequency, particularly at client level. Also, talking about interests can be a sensitive issue. Some respondents mentioned that the guaranteed anonymity and member check assured them to talk more freely about interests, in order to allow for solid research and proper practical implications. Other respondents were more careful in choosing their words and explicitly mentioned that they were doing this. Moreover, purposeful sampling based on the SNA results, led to a diverse group of interviewees, which made it possible to reflect on poorly to highly connected positions of different kinds of organisations and the role interests have on the position of a certain organisation. However, in a more ideal situation more medical organisations would have been interviewed, as they are currently underrepresented. Although general practitioners are important stakeholders in organising good care for people with dementia, GP-practices in Twente are represented in the network by an overarching body: the GP care groups. We were not able to conclude what consequence this has on network dynamics and the ability to reach the network goal. Hence, we propose further research on these questions and on how GP’s are involved in other regional IDC-networks. This could clarify which way of involvement (direct or indirect via care groups) is more beneficial for organising integrated dementia care in the region.

Carrying out a focus group, led to the realisation that our overall findings are likely to be generalisable to other IDC-networks in the Netherlands – like the role of network coordinators and the finding that highly connected organisations are often care organisations which provide case management. However, as the IDC-network ‘Dementie Twente’ is known as a relatively active network in a collaborative region, selection bias limits our ability to generalise specific findings with regards to the structure of IDC-networks and the specific role interests play on network relationships. This requires further research on more IDC-networks. When this follow-up research will use the same explanatory mixed methods design, a thick understanding of the network structure and the internal processes of collaboration can be acquired and compared with this single case study. Additional efforts are recommended to ensure full response to SNA-questionnaires, in order to generate fully developed Social Network Graphs. As interorganisational care networks with network coordinators are regularly used governance mechanisms to tackle wicked health problems like dementia care, such studies can give insights in how to implement these networks properly, adapted to the context in which they operate, so that the working mechanisms of integrated care models translate to the provision of effective (dementia) care.

## Conclusions

It can be concluded that organisational and personal interests have a considerable influence on the participation of organisations in an IDC-network. Especially conflicting interests (e.g., financial interests and clashing visions) between organisations were found to have a significant influence on how a partner is positioned in the network. Because conflicting interests lead to less-optimal cooperation at organisational level, these are likely to stand in the way of reaching the collective goal of the IDC-network: optimising the wellbeing of people with dementia and their informal caregivers in the region. Thus, the network coordinator should act to resolve disputes arising from conflicting interests. In order to be able to function as mediator, impartiality of the network coordinator is of great importance. More importantly, a strategy is needed to not only resolve, but to also prevent (conflicting) interests to hamper cooperation in an IDC-network. We recommend that this strategy should centre around focusing on one clear shared common goal which is present at all levels of integration, i.e., improving the wellbeing of the person with dementia and his/her caregiver. Only with this main focus and smooth cooperation, dementia care can be pushed to a higher level.

## Additional Files

The additional files for this article are as follows:

10.5334/ijic.6424.s1 Appendix 1. The Dutch (dementia) policy context.

10.5334/ijic.6424.s2 Appendix 2. DoFile SNA in R.

10.5334/ijic.6424.s3 Appendix 3. SNA Results information-exchange network.

10.5334/ijic.6424.s4 Appendix 4. SNA Results organisational cooperation network.

10.5334/ijic.6424.s5 Appendix 5. SNA Results cooperation at client level network.
