# Supplementary material for: One for All, All for One: A Mixed Methods Case Study into the Role Organisational and Personal Interests Play on Cooperation in Dutch Integrated Dementia Care Networks
Source: Int J Integr Care. 2022 Aug 17;22(3):10. doi: 10.5334/ijic.6424 (PMC9389949; doi:10.5334/ijic.6424)
Supplement: Appendix 1. — The Dutch (dementia) policy context. [file ijic-22-3-6424-s1.pdf]

Appendix 1 The Dutch (dementia) policy context

During the early stages of living with dementia, care is mostly provided by primary care practitioners, informal caregivers and patient federations. Hospital's specialist memory clinics or mental health services carry out the medical diagnostics. After diagnosis, local services decide on the specific care package, including services such as case management, personal care, housekeeping and counselling. If living at home is no longer possible, persons with dementia can move to sheltered housing or nursing homes. The local governments are responsible for purchasing long-term care (Wlz) and social support (Wmo), while the national government purchases medical care (Zvw).

Integrated dementia care (IDC-) networks in the Netherlands

The Dutch government promotes close collaboration between dementia care providers and case managers within regional interorganisational dementia care (IDC-) networks. As many different providers are involved in providing dementia care to a specific person, it is important that different domains - ranging from the care, social and medical domain - and professionals (like specialized nurses, geriatricians, or other medical specialists) are either as a network partner or as stakeholder connected with the IDC-network. In 2011, the 'Deltaplan Dementie' was initiated; this program focused on building regional infrastructures of dementia care networks, containing all relevant stakeholders in the field. Currently there are 60-65 IDC-networks (see figure A) which are all different with respect to the size of the work area, diversity of partners, but also in the way they are organised. Nonetheless, they all have more or less the same overall goal; providing person-centred and integrated care for people with dementia and their caregivers, so that people with dementia are able to live as long as possible at home with as few as possible risks. One of the main tasks of the regional IDC-networks is to organise case management, but they also serve much wider goals as translating national policy to the regional context (e.g. implementing the 'Dementia Care Standard' by 2025). Regular funding for het IDC-networks and their coordination activities is not arranged yet.

Case management

In order to be able to overcome fragmentation and to provide integrated dementia care, case management has been emphasized in the publication of the 'Dementia Care Standard'. The aim is to provide all people with (and suspicion of) dementia and their informal caregivers from the start of the diagnostic trajectory with a fixed coordination and contact point; the case manager. Next to coordinating the care process, the case manager ensures that the needs of people and their caregivers are the centre decisions made in the care process, which requires a more holistic view on health where physical, mental, social and the spiritual dimension of health and quality of life occupy the centre stage. Two prominent dementia case management models exist in the Netherlands[1]: the linkage model and the intensive case management/joint agency model. In the former multiple case management providers are active and the case manager acts as mediator between the client and various care agencies, while in the latter case management and any additional care services are embedded in one independent organization.

---

[1] MacNeil Vroomen J, Van Mierlo LD, Van de Ven PM, Bosmans JE, Van den Dungen P, Meiland FJM, Dröes RM, van Charante EP M, Van der Horst HE, De Rooij SE, Van Hout HPJ: Comparing Dutch Case management care models for people with dementia and their caregivers: The design of the COMPAS study. BMC Health Serv Res 2012, 12:132

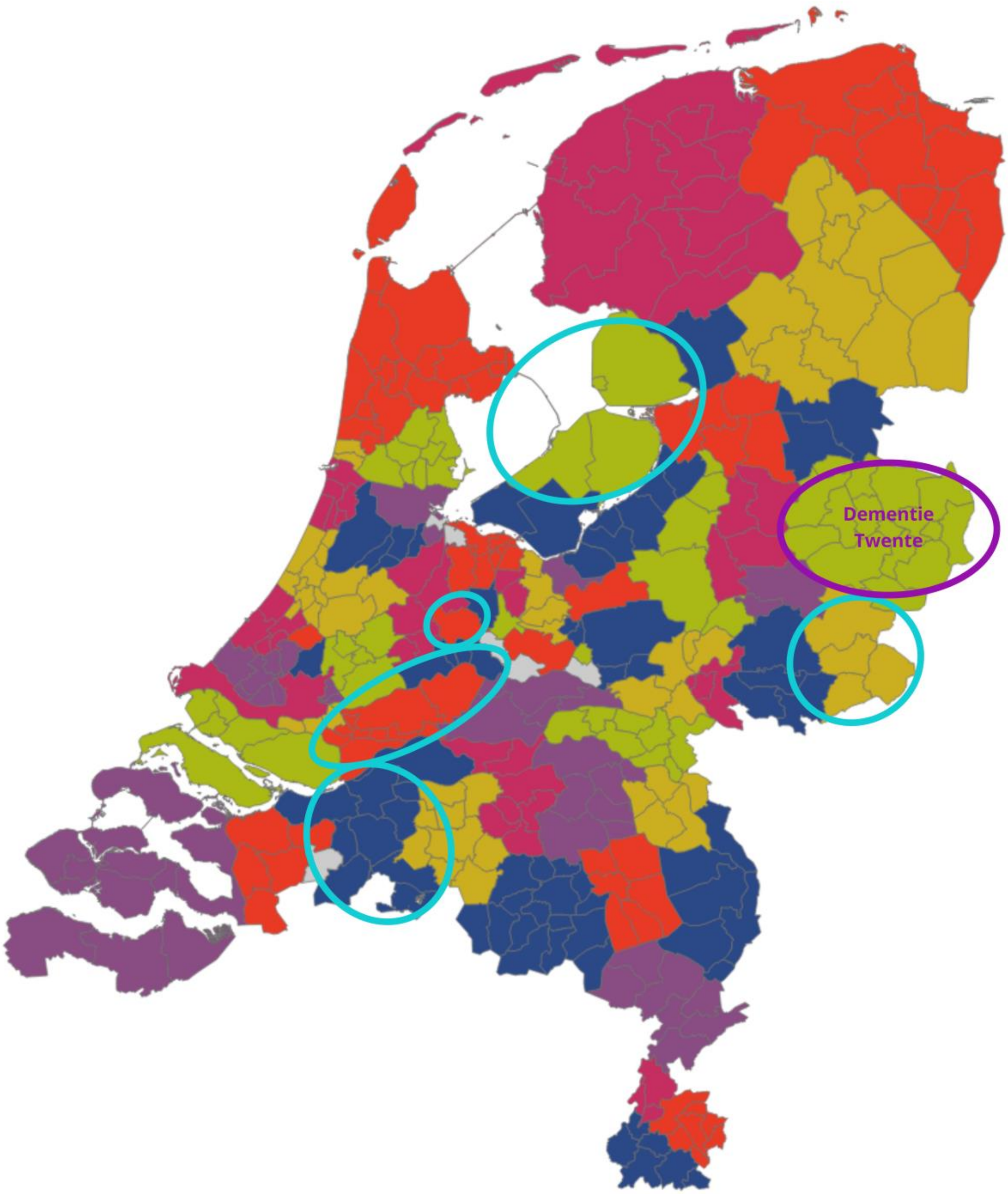

Figuur A: An overview of the operating areas of 60-65 IDC networks in the Netherlands and the IDC-networks of the network coordinators which participated in the focus group (circled in blue and purple). Source: https://www.dementienetwerknederland.nl/de-netwerken-dementie-in-nederland/netwerken-dementie-in-nederland/
