# Supplementary material for: One for All, All for One: A Mixed Methods Case Study into the Role Organisational and Personal Interests Play on Cooperation in Dutch Integrated Dementia Care Networks
Source: Int J Integr Care. 2022 Aug 17;22(3):10. doi: 10.5334/ijic.6424 (PMC9389949; doi:10.5334/ijic.6424)
Supplement: Appendix 2. — DoFile SNA in R. [file ijic-22-3-6424-s2.pdf]

Appendix 2 DoFile SNA in R

#step0loadpackages

```
install.packages('EconGeo')
install.packages('igraph')
install.packages('DT')
install.packages('htmlTable')
install.packages('networkD3')
library(EconGeo)
library(igraph)
library(DT)
library(htmlTable)
library(networkD3)
install.packages('sna')
install.packages('ndtv')
install.packages('visNetwork')
install.packages('RColorBrewer')
library('RColorBrewer')
install.packages('extrafont')
library('extrafont')
```

#step1 load edgelists & load node list

```
nodes = Nodelist
links = ELI2AN
head(nodes)
head(links)
```

#step2 create graph g

```
g <-graph_from_data_frame(d=links, directed=TRUE, vertices=nodes)
```

#step3 simplify graph

```
g <- simplify(g, remove.multiple = F, remove.loops = T)
as_edgelist(g, names=T)
```

#step4 format graph

```
pal <- brewer.pal(length(unique(V(g)$Soort)), "Pastel1")
plot(g, edge.arrow.size=.2, edge.color="grey",  vertex.color = pal[as.numeric(as.factor(vertex_attr(g, "Soort")))],
vertex.frame.color="#ffffff",
     vertex.label=V(g)$Code, vertex.label.color="black" )
```

#step5 interactive graph

```
simpleNetwork(links, linkDistance=30, fontSize = 7, opacity = 0.7, zoom = T)
```

#step5 Network-level metrics

```
networkdata = data.frame(
  dens = graph.density(g),
```

```
  reci = reciprocity(g),
  avpl = average.path.length(g),
  tran = transitivity(g),
  gini = Gini(g))

networkdata
netable <- htmlTable(networkdata, header=c("Density", "Reciprocity", "Path length",
                "Transitivity", "Gini"), rnames=FALSE, align="left")
Netable
```

#step6 Node-level metrics

```
nodedata = data.frame(
  dgr = degree(g, mode=c("all"), loops=FALSE),
  ind = degree(g, mode=c("in"), loops=FALSE),
  oud = degree(g, mode=c("out"), loops=FALSE),
  bc = betweenness(g),
  cc = closeness(g),
  ec = evcent(g)$vector)
nodedata
notable <- htmlTable(nodedata, header=c("Degree", "Indegree", "Outdegree",
                 "Betweenness", "Closeness","Eigenvector Centrality" ), rnames=V(g)$Code,
                 align="right")
notable
```
