# Supplementary material for: One for All, All for One: A Mixed Methods Case Study into the Role Organisational and Personal Interests Play on Cooperation in Dutch Integrated Dementia Care Networks
Source: Int J Integr Care. 2022 Aug 17;22(3):10. doi: 10.5334/ijic.6424 (PMC9389949; doi:10.5334/ijic.6424)
Supplement: Appendix 3. — SNA Results information-exchange network. [file ijic-22-3-6424-s3.pdf]

**Appendix 3 SNA results information exchange network**

Informational Network Metrics

The SNA (n= 25) reveals that 30% of the possible ties between organisations are present on the frequency level of 'occasionally or more frequent' exchange of information, this percentage drops to 13% on the 'regularly or more frequent level' and to 4% on the frequency level 'very often'. Half of the ties on the lowest level are reciprocal and this percentage, likewise, drops when the frequency levels of information exchange increase. The average path length across the frequency levels is between 1.5 and 2. Lastly, the degree measures per node are more evenly distributed on a lower than higher level of information exchange frequency (Gini coefficient).

Level 1: Occasionally or higher

| Density | Reciprocity | Path length | Transitivity | Gini |
|---|---|---|---|---|
| 0.31 | 0.52 | 1.55 | 0.63 | 0.68 |

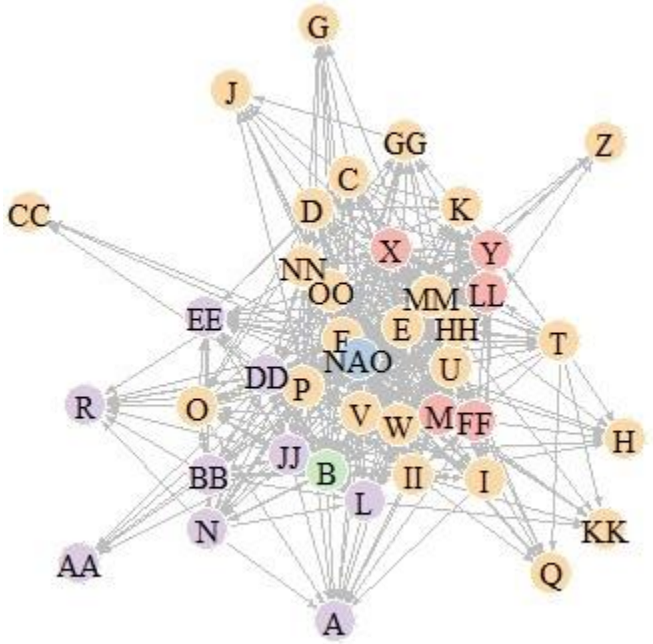


| | |
|---|---|
| Yellow | Care organisations |
| Purple | Social welfare organisations |
| Red | Medical organisations |
| Blue | Network Coordinator |
| Green | Patient organisation |

*Table 8.2 Legend Network Graph Colours*

Level 2: Regularly or higher

| Density | Reciprocity | Path length | Transitivity | Gini |
|---|---|---|---|---|
| 0.13 | 0.32 | 2.11 | 0.37 | 0.95 |

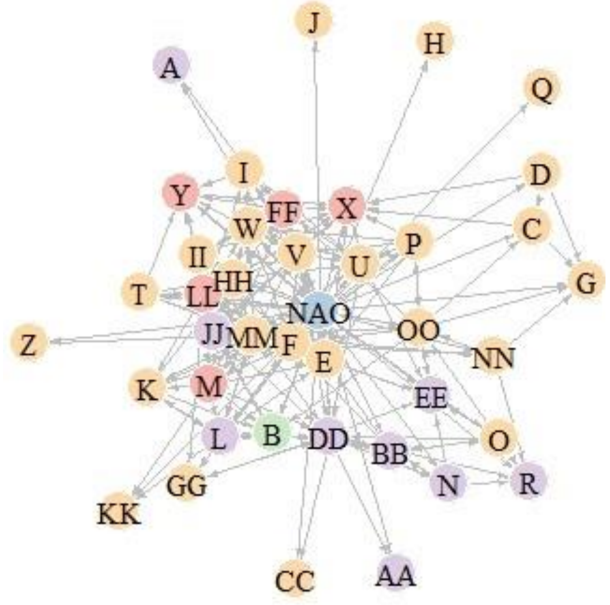


Level 3: Very often

| Density | Reciprocity | Path length | Transitivity | Gini |
|---|---|---|---|---|
| 0.04 | 0.22 | 2.06 | 0.31 | - |

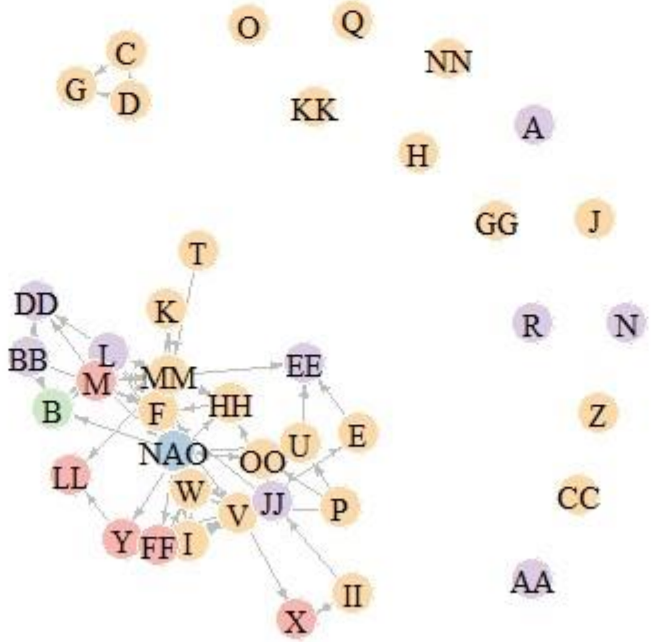

Level 1: Occasionally or higher

| | Degree | Indegree | Outdegree | Betweenness | Closeness | Eigenvector Centrality |
|---|---|---|---|---|---|---|
| NAO | 67 | 27 | 40 | 159.301 | 0.025 | 1.000 |
| F | 63 | 24 | 39 | 120.637 | 0.024 | 0.950 |
| MM | 49 | 19 | 30 | 54.737 | 0.020 | 0.783 |
| V | 35 | 16 | 19 | 10.377 | 0.016 | 0.684 |
| OO | 38 | 21 | 17 | 23.276 | 0.016 | 0.680 |
| E | 38 | 16 | 22 | 24.101 | 0.017 | 0.675 |
| U | 37 | 16 | 21 | 25.389 | 0.017 | 0.670 |
| W | 34 | 16 | 18 | 13.317 | 0.016 | 0.646 |
| DD | 39 | 17 | 22 | 34.438 | 0.017 | 0.645 |
| II | 36 | 13 | 23 | 18.537 | 0.018 | 0.641 |
| P | 34 | 13 | 21 | 15.452 | 0.017 | 0.627 |
| JJ | 35 | 13 | 22 | 16.685 | 0.017 | 0.618 |
| B | 34 | 20 | 14 | 21.475 | 0.015 | 0.609 |
| NN | 32 | 11 | 21 | 10.850 | 0.017 | 0.596 |
| LL | 31 | 16 | 15 | 12.499 | 0.015 | 0.581 |
| FF | 30 | 13 | 17 | 11.272 | 0.016 | 0.562 |
| Y | 24 | 18 | 6 | 2.094 | 0.014 | 0.477 |
| C | 25 | 8 | 17 | 3.431 | 0.016 | 0.461 |
| L | 25 | 11 | 14 | 8.694 | 0.015 | 0.455 |
| D | 24 | 6 | 18 | 3.709 | 0.016 | 0.437 |
| HH | 23 | 23 | 0 | 0.000 | 0.001 | 0.423 |
| I | 21 | 10 | 11 | 2.058 | 0.014 | 0.422 |
| M | 22 | 7 | 15 | 4.785 | 0.015 | 0.416 |
| X | 21 | 21 | 0 | 0.000 | 0.001 | 0.401 |
| K | 19 | 11 | 8 | 2.349 | 0.014 | 0.379 |
| BB | 21 | 8 | 13 | 3.396 | 0.015 | 0.377 |
| O | 20 | 8 | 12 | 5.164 | 0.015 | 0.367 |
| T | 20 | 5 | 15 | 1.177 | 0.015 | 0.365 |
| N | 18 | 6 | 12 | 1.348 | 0.015 | 0.346 |
| GG | 14 | 8 | 6 | 1.452 | 0.014 | 0.296 |
| EE | 16 | 16 | 0 | 0.000 | 0.001 | 0.295 |
| A | 13 | 13 | 0 | 0.000 | 0.001 | 0.231 |
| R | 9 | 9 | 0 | 0.000 | 0.001 | 0.172 |
| J | 8 | 8 | 0 | 0.000 | 0.001 | 0.163 |
| Q | 7 | 7 | 0 | 0.000 | 0.001 | 0.158 |
| H | 7 | 7 | 0 | 0.000 | 0.001 | 0.153 |
| G | 7 | 7 | 0 | 0.000 | 0.001 | 0.153 |
| KK | 7 | 7 | 0 | 0.000 | 0.001 | 0.143 |
| Z | 5 | 5 | 0 | 0.000 | 0.001 | 0.127 |
| AA | 5 | 5 | 0 | 0.000 | 0.001 | 0.112 |
| CC | 3 | 3 | 0 | 0.000 | 0.001 | 0.081 |

Level 2: Regularly or higher

| | Degree | Indegree | Outdegree | Betweenness | Closeness | Eigenvector Centrality |
|---|---|---|---|---|---|---|
| NAO | 67 | 27 | 40 | 159.301 | 0.025 | 1.000 |
| F | 63 | 24 | 39 | 120.637 | 0.024 | 0.950 |
| MM | 49 | 19 | 30 | 54.737 | 0.020 | 0.783 |
| V | 35 | 16 | 19 | 10.377 | 0.016 | 0.684 |
| OO | 38 | 21 | 17 | 23.276 | 0.016 | 0.680 |
| E | 38 | 16 | 22 | 24.101 | 0.017 | 0.675 |
| U | 37 | 16 | 21 | 25.389 | 0.017 | 0.670 |
| W | 34 | 16 | 18 | 13.317 | 0.016 | 0.646 |
| DD | 39 | 17 | 22 | 34.438 | 0.017 | 0.645 |
| II | 36 | 13 | 23 | 18.537 | 0.018 | 0.641 |
| P | 34 | 13 | 21 | 15.452 | 0.017 | 0.627 |
| JJ | 35 | 13 | 22 | 16.685 | 0.017 | 0.618 |
| B | 34 | 20 | 14 | 21.475 | 0.015 | 0.609 |
| NN | 32 | 11 | 21 | 10.850 | 0.017 | 0.596 |
| LL | 31 | 16 | 15 | 12.499 | 0.015 | 0.581 |
| FF | 30 | 13 | 17 | 11.272 | 0.016 | 0.562 |
| Y | 24 | 18 | 6 | 2.094 | 0.014 | 0.477 |
| C | 25 | 8 | 17 | 3.431 | 0.016 | 0.461 |
| L | 25 | 11 | 14 | 8.694 | 0.015 | 0.455 |
| D | 24 | 6 | 18 | 3.709 | 0.016 | 0.437 |
| HH | 23 | 23 | 0 | 0.000 | 0.001 | 0.423 |
| I | 21 | 10 | 11 | 2.058 | 0.014 | 0.422 |
| M | 22 | 7 | 15 | 4.785 | 0.015 | 0.416 |
| X | 21 | 21 | 0 | 0.000 | 0.001 | 0.401 |
| K | 19 | 11 | 8 | 2.349 | 0.014 | 0.379 |
| BB | 21 | 8 | 13 | 3.396 | 0.015 | 0.377 |
| O | 20 | 8 | 12 | 5.164 | 0.015 | 0.367 |
| T | 20 | 5 | 15 | 1.177 | 0.015 | 0.365 |
| N | 18 | 6 | 12 | 1.348 | 0.015 | 0.346 |
| GG | 14 | 8 | 6 | 1.452 | 0.014 | 0.296 |
| EE | 16 | 16 | 0 | 0.000 | 0.001 | 0.295 |
| A | 13 | 13 | 0 | 0.000 | 0.001 | 0.231 |
| R | 9 | 9 | 0 | 0.000 | 0.001 | 0.172 |
| J | 8 | 8 | 0 | 0.000 | 0.001 | 0.163 |
| Q | 7 | 7 | 0 | 0.000 | 0.001 | 0.158 |
| H | 7 | 7 | 0 | 0.000 | 0.001 | 0.153 |
| G | 7 | 7 | 0 | 0.000 | 0.001 | 0.153 |
| KK | 7 | 7 | 0 | 0.000 | 0.001 | 0.143 |
| Z | 5 | 5 | 0 | 0.000 | 0.001 | 0.127 |
| AA | 5 | 5 | 0 | 0.000 | 0.001 | 0.112 |
| CC | 3 | 3 | 0 | 0.000 | 0.001 | 0.081 |

Level 3: Very often

| | Degree | Indegree | Outdegree | Betweenness | Closeness | Eigenvector Centrality |
|---|---|---|---|---|---|---|
| NAO | 15 | 5 | 10 | 85.167 | 0.001 | 1.000 |
| MM | 13 | 4 | 9 | 55.250 | 0.001 | 0.786 |
| I | 8 | 3 | 5 | 20.500 | 0.001 | 0.687 |
| V | 11 | 4 | 7 | 30.500 | 0.001 | 0.636 |
| L | 7 | 2 | 5 | 4.667 | 0.001 | 0.533 |
| M | 7 | 1 | 6 | 4.667 | 0.001 | 0.533 |
| W | 6 | 6 | 0 | 0.000 | 0.001 | 0.508 |
| FF | 5 | 2 | 3 | 0.000 | 0.001 | 0.477 |
| F | 6 | 6 | 0 | 0.000 | 0.001 | 0.374 |
| Y | 4 | 3 | 1 | 5.000 | 0.001 | 0.335 |
| B | 4 | 4 | 0 | 0.000 | 0.001 | 0.295 |
| OO | 3 | 2 | 1 | 8.500 | 0.001 | 0.293 |
| U | 4 | 3 | 1 | 3.500 | 0.001 | 0.265 |
| HH | 3 | 3 | 0 | 0.000 | 0.001 | 0.264 |
| P | 3 | 0 | 3 | 0.000 | 0.001 | 0.162 |
| JJ | 4 | 1 | 3 | 3.000 | 0.001 | 0.160 |
| DD | 3 | 3 | 0 | 0.000 | 0.001 | 0.160 |
| EE | 3 | 3 | 0 | 0.000 | 0.001 | 0.157 |
| LL | 2 | 2 | 0 | 0.000 | 0.001 | 0.152 |
| BB | 3 | 0 | 3 | 0.000 | 0.001 | 0.112 |
| E | 2 | 1 | 1 | 1.250 | 0.001 | 0.107 |
| K | 1 | 1 | 0 | 0.000 | 0.001 | 0.107 |
| T | 1 | 0 | 1 | 0.000 | 0.001 | 0.107 |
| X | 2 | 2 | 0 | 0.000 | 0.001 | 0.091 |
| II | 2 | 0 | 2 | 0.000 | 0.001 | 0.034 |
| G | 2 | 2 | 0 | 0.000 | 0.001 | 0.000 |
| D | 2 | 0 | 2 | 0.000 | 0.001 | 0.000 |
| C | 2 | 1 | 1 | 0.000 | 0.001 | 0.000 |
| A | 0 | 0 | 0 | 0.000 | 0.001 | 0.000 |
| H | 0 | 0 | 0 | 0.000 | 0.001 | 0.000 |
| J | 0 | 0 | 0 | 0.000 | 0.001 | 0.000 |
| N | 0 | 0 | 0 | 0.000 | 0.001 | 0.000 |
| O | 0 | 0 | 0 | 0.000 | 0.001 | 0.000 |
| Q | 0 | 0 | 0 | 0.000 | 0.001 | 0.000 |
| R | 0 | 0 | 0 | 0.000 | 0.001 | 0.000 |
| Z | 0 | 0 | 0 | 0.000 | 0.001 | 0.000 |
| AA | 0 | 0 | 0 | 0.000 | 0.001 | 0.000 |
| CC | 0 | 0 | 0 | 0.000 | 0.001 | 0.000 |
| GG | 0 | 0 | 0 | 0.000 | 0.001 | 0.000 |
| KK | 0 | 0 | 0 | 0.000 | 0.001 | 0.000 |
| NN | 0 | 0 | 0 | 0.000 | 0.001 | 0.000 |
