# Supplementary material for: One for All, All for One: A Mixed Methods Case Study into the Role Organisational and Personal Interests Play on Cooperation in Dutch Integrated Dementia Care Networks
Source: Int J Integr Care. 2022 Aug 17;22(3):10. doi: 10.5334/ijic.6424 (PMC9389949; doi:10.5334/ijic.6424)
Supplement: Appendix 4. — SNA Results organisational cooperation network. [file ijic-22-3-6424-s4.pdf]

Appendix 4 SNA results organisational cooperation network

Organisation-level Network Metrics

The SNA (n= 23) shows that 21% of the possible cooperation ties between organisations are present on the frequency level ‘occasionally or more frequent’. This percentage drops sharply as we go to lower levels of cooperation frequency: 9.6% of the possible ties are present of the ‘regularly of more frequent’ cooperation level and 5.3% of the possible ties on the ‘very often’ level. Also the percentage of reciprocal ties drops as one moves higher up in the frequency levels: from 21.4% to 2.3%. The average path length differs not a lot across all levels and fluctuates between 1.6 and 1.9. Also, the degree measures per node are more evenly distributed on a lower than higher level of information exchange

Density	Reciprocity	Path length	Gini
0.10	0.32	1.89	0.95

Level 1: Occasionally or higher

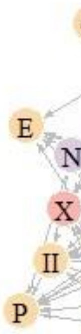

Level 2: Regularly or higher

Density	Reciprocity	Path length	Gini
0.21	0.46	1.66	0.81

Density	Reciprocity	Path length	Gini
0.02	0.05	1.87	-

Level 3: Very often

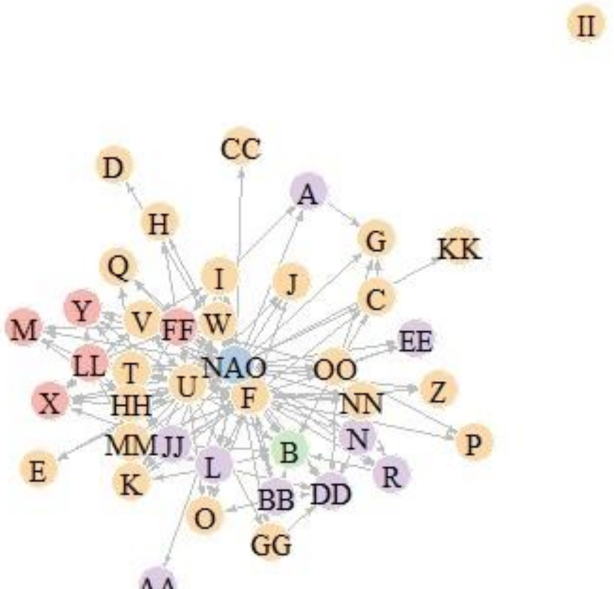

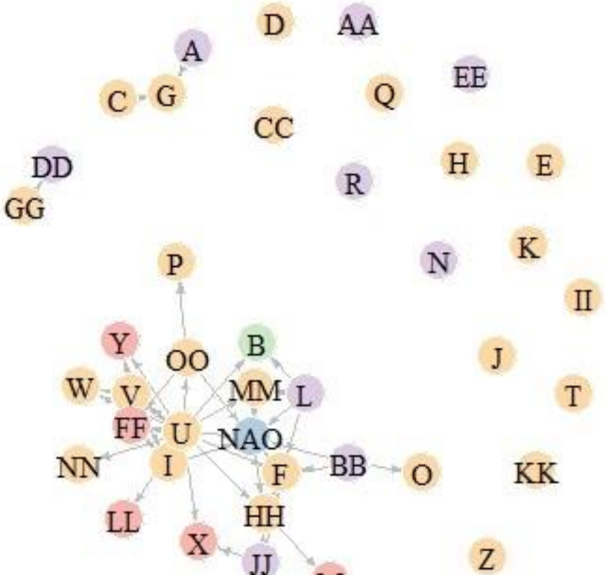

Organisation-level Node Metrics

Level 1: Occasionally or higher

	**Degree**	**Indegree**	**Outdegree**	**Betweenness**	**Closeness**	**Eigenvector Centrality**
NAO	63	23	40	317.211	0.025	1.000
F	52	18	34	118.358	0.022	0.907
U	39	15	24	77.854	0.018	0.767
W	31	12	19	13.179	0.016	0.694
B	33	12	21	23.828	0.017	0.688
T	43	3	40	9.944	0.025	0.661
JJ	32	11	21	22.222	0.017	0.640
MM	30	12	18	23.865	0.016	0.602
V	25	13	12	5.448	0.015	0.600
FF	26	11	15	11.099	0.015	0.583
OO	29	11	18	18.259	0.016	0.576
LL	22	12	10	6.622	0.014	0.533
I	19	10	9	2.870	0.014	0.463
Y	17	12	5	1.204	0.013	0.450
NN	17	7	10	1.885	0.014	0.445
L	16	8	8	2.367	0.014	0.383
HH	16	16	0	0.000	0.001	0.376
N	15	4	11	0.803	0.014	0.374
X	15	15	0	0.000	0.001	0.368
GG	15	5	10	2.725	0.014	0.358
K	13	7	6	0.533	0.014	0.349
II	13	7	6	0.712	0.014	0.335
BB	13	5	8	0.636	0.014	0.309
DD	12	12	0	0.000	0.001	0.286
A	10	8	2	1.833	0.013	0.263
E	9	9	0	0.000	0.001	0.228
P	9	8	1	0.375	0.011	0.220
EE	7	7	0	0.000	0.001	0.197
O	7	7	0	0.000	0.001	0.191
C	7	5	2	0.167	0.013	0.190
J	6	6	0	0.000	0.001	0.166
M	6	6	0	0.000	0.001	0.145
H	5	5	0	0.000	0.001	0.143
R	5	5	0	0.000	0.001	0.142
D	4	3	1	0.000	0.013	0.139
KK	4	4	0	0.000	0.001	0.130
G	6	6	0	0.000	0.001	0.128
Q	3	3	0	0.000	0.001	0.100
Z	3	3	0	0.000	0.001	0.100
CC	3	3	0	0.000	0.001	0.100
AA	2	2	0	0.000	0.001	0.065

Level 2: Regularly or higher

	Degree	Indegree	Outdegree	Betweenness	Closeness	Eigenvector Centrality
NAO	46	13	33	342.583	0.012	1.000
F	34	11	23	132.187	0.010	0.905
U	25	5	20	38.570	0.010	0.732
FF	14	6	8	23.124	0.009	0.555
W	11	7	4	8.124	0.009	0.498
I	11	6	5	7.379	0.008	0.478
V	12	6	6	6.278	0.008	0.471
LL	12	3	9	5.503	0.008	0.436
MM	10	5	5	3.136	0.009	0.409
L	10	3	7	2.639	0.009	0.406
T	10	2	8	1.994	0.009	0.403
OO	13	5	8	45.517	0.009	0.402
HH	9	9	0	0.000	0.001	0.359
Y	6	5	1	3.272	0.008	0.284
B	7	6	1	2.704	0.008	0.278
K	6	6	0	0.000	0.001	0.271
JJ	7	1	6	0.450	0.008	0.260
BB	6	1	5	0.611	0.009	0.255
N	5	1	4	0.000	0.009	0.249
DD	7	7	0	0.000	0.001	0.236
NN	5	3	2	0.333	0.007	0.224
X	5	5	0	0.000	0.001	0.204
O	4	4	0	0.000	0.001	0.203
J	3	3	0	0.000	0.001	0.185
C	4	3	1	0.736	0.001	0.171
Q	3	3	0	0.000	0.001	0.162
EE	3	3	0	0.000	0.001	0.162
R	3	3	0	0.000	0.001	0.150
M	3	3	0	0.000	0.001	0.140
GG	3	2	1	0.250	0.001	0.138
Z	2	2	0	0.000	0.001	0.134
E	2	2	0	0.000	0.001	0.122
H	2	2	0	0.000	0.001	0.122
G	4	4	0	0.000	0.001	0.119
A	3	2	1	0.611	0.001	0.112
P	2	2	0	0.000	0.001	0.092
D	1	1	0	0.000	0.001	0.070
AA	1	1	0	0.000	0.001	0.070
CC	1	1	0	0.000	0.001	0.070
KK	1	1	0	0.000	0.001	0.070
II	0	0	0	0.000	0.001	0.000

Level 3: Very often

	Degree	Indegree	Outdegree	Betweenness	Closeness	Eigenvector Centrality
U	15	1	14	26.500	0.001	1.000
V	6	2	4	22.000	0.001	0.664
I	4	3	1	1.500	0.001	0.485
NAO	6	6	0	0.000	0.001	0.478
FF	4	2	2	12.000	0.001	0.469
OO	4	1	3	3.000	0.001	0.375
MM	3	2	1	0.000	0.001	0.320
W	2	2	0	0.000	0.001	0.309
Y	2	2	0	0.000	0.001	0.309
F	4	4	0	0.000	0.001	0.283
L	4	0	4	0.000	0.001	0.244
B	2	2	0	0.000	0.001	0.231
X	2	2	0	0.000	0.001	0.210
HH	2	2	0	0.000	0.001	0.210
LL	1	1	0	0.000	0.001	0.186
NN	1	1	0	0.000	0.001	0.186
BB	3	0	3	0.000	0.001	0.146
JJ	3	0	3	0.000	0.001	0.131
P	1	1	0	0.000	0.001	0.070
O	1	1	0	0.000	0.001	0.027
DD	1	1	0	0.000	0.001	0.000
GG	1	0	1	0.000	0.001	0.000
A	1	0	1	0.000	0.001	0.000
C	1	0	1	0.000	0.001	0.000
G	2	2	0	0.000	0.001	0.000
D	0	0	0	0.000	0.001	0.000
E	0	0	0	0.000	0.001	0.000
H	0	0	0	0.000	0.001	0.000
J	0	0	0	0.000	0.001	0.000
K	0	0	0	0.000	0.001	0.000
M	0	0	0	0.000	0.001	0.000
N	0	0	0	0.000	0.001	0.000
Q	0	0	0	0.000	0.001	0.000
R	0	0	0	0.000	0.001	0.000
T	0	0	0	0.000	0.001	0.000
Z	0	0	0	0.000	0.001	0.000
AA	0	0	0	0.000	0.001	0.000
CC	0	0	0	0.000	0.001	0.000
EE	0	0	0	0.000	0.001	0.000
II	0	0	0	0.000	0.001	0.000
KK	0	0	0	0.000	0.001	0.000
