# Supplementary material for: One for All, All for One: A Mixed Methods Case Study into the Role Organisational and Personal Interests Play on Cooperation in Dutch Integrated Dementia Care Networks
Source: Int J Integr Care. 2022 Aug 17;22(3):10. doi: 10.5334/ijic.6424 (PMC9389949; doi:10.5334/ijic.6424)
Supplement: Appendix 5. — SNA Results cooperation at client level network. [file ijic-22-3-6424-s5.pdf]

Appendix 5 SNA results cooperation at client level network

Client-level Network Metrics

The SNA (n= 23) reveals that 26.8% of the possible cooperation ties on client level between organisations are present on the frequency level ‘occasionally or more frequent’. This percentage drops as we go to lower levels of cooperation frequency: 10% of the possible ties are present of the ‘regularly of more frequent’ cooperation level and 4.6% of the possible ties on the ‘very often’ level. Also the percentage of reciprocal ties drops as we move higher into the frequency levels: from 35% to 5.3%. The average path length fluctuates between 1.57 and 2 across the frequency levels. Also, the degree measures per node are more evenly distributed on a lower than higher level of information exchange frequency.

Level 1: Occasionally or higher

Density	Reciprocity	Path length	Gini
0.27	0.35	1.57	0.44

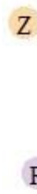

Level 2: Regularly or higher

Density	Reciprocity	Path length	Gini
0.10	0.12	2.19	0.66

Level 3: Very often

Density	Reciprocity	Path length	Gini
0.04	0.05	1.99	0.88

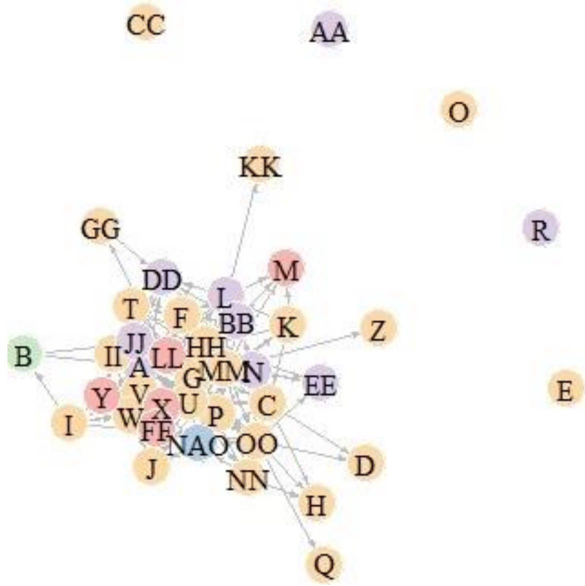

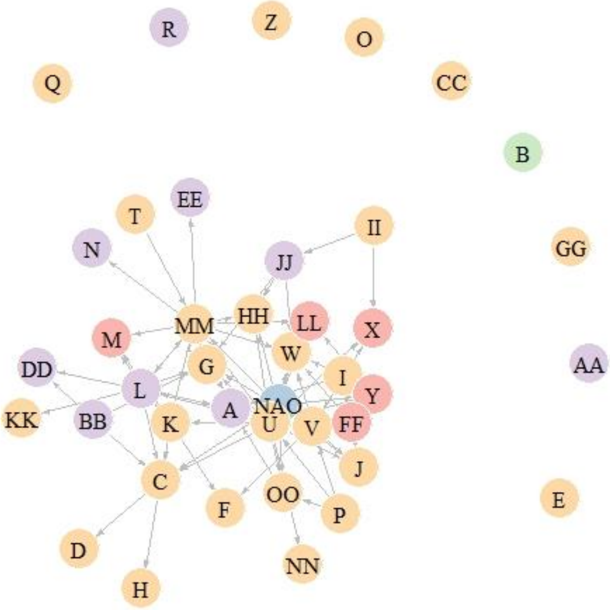

Client-level Node Metrics

Level 1: Occasionally or higher

	Degree	Indegree	Outdegree	Betweenness	Closeness	Eigenvector Centrality
F	53	15	38	111.700	0.024	1.000
MM	46	16	30	90.378	0.020	0.911
U	41	15	26	52.819	0.019	0.881
NAO	45	6	39	36.494	0.024	0.794
JJ	37	13	24	33.577	0.018	0.785
W	33	15	18	15.277	0.016	0.781
V	33	14	19	12.077	0.016	0.779
OO	34	17	17	25.359	0.016	0.754
C	34	16	18	26.371	0.016	0.753
NN	32	11	21	17.812	0.017	0.727
FF	31	14	17	18.518	0.016	0.710
II	33	10	23	21.267	0.018	0.691
LL	29	14	15	15.234	0.015	0.679
P	29	8	21	14.828	0.017	0.654
Y	21	15	6	1.673	0.012	0.534
A	23	23	0	0.000	0.001	0.518
D	21	6	15	3.229	0.015	0.511
L	23	9	14	11.098	0.015	0.510
HH	20	20	0	0.000	0.001	0.483
I	19	8	11	2.116	0.014	0.476
X	18	18	0	0.000	0.001	0.453
G	20	20	0	0.000	0.001	0.450
B	22	9	13	13.668	0.014	0.436
T	20	5	15	2.679	0.015	0.434
K	18	9	9	5.224	0.013	0.430
N	19	7	12	9.871	0.015	0.416
BB	19	6	13	5.282	0.015	0.395
DD	15	15	0	0.000	0.001	0.346
GG	14	8	6	1.447	0.012	0.332
J	11	11	0	0.000	0.001	0.297
Q	11	11	0	0.000	0.001	0.287
EE	10	10	0	0.000	0.001	0.240
M	9	9	0	0.000	0.001	0.202
KK	7	7	0	0.000	0.001	0.186
H	6	6	0	0.000	0.001	0.176
R	5	5	0	0.000	0.001	0.136
O	5	5	0	0.000	0.001	0.130
E	4	4	0	0.000	0.001	0.112
AA	4	4	0	0.000	0.001	0.106
Z	3	3	0	0.000	0.001	0.096
CC	3	3	0	0.000	0.001	0.095

Level 2: Regularly or higher

	Degree	Indegree	Outdegree	Betweenness	Closeness	Eigenvector Centrality
U	24	7	17	149.321	0.002	1.000
MM	23	7	16	108.376	0.002	0.847
V	17	7	10	28.754	0.002	0.757
W	16	10	6	58.260	0.002	0.751
LL	18	5	13	64.917	0.002	0.722
FF	16	7	9	47.984	0.002	0.719
G	13	13	0	0.000	0.001	0.539
A	14	14	0	0.000	0.001	0.535
NAO	12	2	10	38.103	0.002	0.518
Y	11	8	3	7.680	0.002	0.498
OO	15	7	8	54.517	0.002	0.492
II	11	0	11	0.000	0.003	0.463
L	12	4	8	29.543	0.002	0.445
J	7	7	0	0.000	0.001	0.409
HH	9	9	0	0.000	0.001	0.401
X	9	9	0	0.000	0.001	0.401
JJ	11	4	7	35.583	0.002	0.400
F	9	8	1	0.560	0.001	0.397
P	8	1	7	22.611	0.002	0.349
I	7	0	7	0.000	0.003	0.335
T	7	0	7	0.000	0.003	0.303
C	10	7	3	19.986	0.001	0.295
N	8	3	5	49.167	0.002	0.295
NN	6	2	4	11.583	0.002	0.268
K	6	1	5	3.333	0.002	0.239
DD	7	7	0	0.000	0.001	0.203
BB	7	1	6	20.556	0.002	0.201
EE	3	3	0	0.000	0.001	0.126
M	3	3	0	0.000	0.001	0.118
B	3	1	2	0.333	0.002	0.098
H	3	3	0	0.000	0.001	0.081
GG	2	1	1	0.833	0.001	0.071
Z	1	1	0	0.000	0.001	0.065
D	2	2	0	0.000	0.001	0.061
Q	1	1	0	0.000	0.001	0.038
KK	1	1	0	0.000	0.001	0.034
E	0	0	0	0.000	0.001	0.000
O	0	0	0	0.000	0.001	0.000
R	0	0	0	0.000	0.001	0.000
AA	0	0	0	0.000	0.001	0.000
CC	0	0	0	0.000	0.001	0.000

Level 3: Very often

	Degree	Indegree	Outdegree	Betweenness	Closeness	Eigenvector Centrality
U	19	3	16	69.000	0.001	1.000
MM	13	4	9	41.167	0.001	0.633
V	12	5	7	45.833	0.001	0.726
NAO	10	0	10	0.000	0.002	0.644
L	10	3	7	33.667	0.001	0.507
W	7	7	0	0.000	0.001	0.506
C	7	5	2	22.000	0.001	0.335
FF	6	3	3	0.500	0.001	0.457
G	6	6	0	0.000	0.001	0.403
Y	5	4	1	2.333	0.001	0.378
A	5	5	0	0.000	0.001	0.353
I	5	0	5	0.000	0.001	0.311
J	4	4	0	0.000	0.001	0.364
HH	4	4	0	0.000	0.001	0.314
OO	4	3	1	0.833	0.001	0.290
K	4	1	3	1.667	0.001	0.260
JJ	4	1	3	3.000	0.001	0.164
P	3	0	3	0.000	0.001	0.259
LL	3	3	0	0.000	0.001	0.259
X	3	3	0	0.000	0.001	0.229
M	3	3	0	0.000	0.001	0.180
BB	3	0	3	0.000	0.001	0.105
DD	2	2	0	0.000	0.001	0.079
II	2	0	2	0.000	0.001	0.051
NN	1	1	0	0.000	0.001	0.129
F	1	1	0	0.000	0.001	0.093
N	1	1	0	0.000	0.001	0.081
T	1	0	1	0.000	0.001	0.081
EE	1	1	0	0.000	0.001	0.081
KK	1	1	0	0.000	0.001	0.065
D	1	1	0	0.000	0.001	0.043
H	1	1	0	0.000	0.001	0.043
B	0	0	0	0.000	0.001	0.000
E	0	0	0	0.000	0.001	0.000
O	0	0	0	0.000	0.001	0.000
Q	0	0	0	0.000	0.001	0.000
R	0	0	0	0.000	0.001	0.000
Z	0	0	0	0.000	0.001	0.000
AA	0	0	0	0.000	0.001	0.000
CC	0	0	0	0.000	0.001	0.000
GG	0	0	0	0.000	0.001	0.000
